# Adaptive Personalized Training Games for Individual and Collaborative Rehabilitation of People with Multiple Sclerosis

**DOI:** 10.1155/2014/345728

**Published:** 2014-05-28

**Authors:** Johanna Renny Octavia, Karin Coninx

**Affiliations:** ^1^Industrial Engineering Department, Parahyangan Catholic University, Ciumbuleuit 94, Bandung 40141, Indonesia; ^2^Expertise Centre for Digital Media-tUL-iMinds, Hasselt University, Wetenschapspark 2, 3590 Diepenbeek, Belgium

## Abstract

Any rehabilitation involves people who are unique individuals with their own characteristics and rehabilitation needs, including patients suffering from Multiple Sclerosis (MS). The prominent variation of MS symptoms and the disease severity elevate a need to accommodate the patient diversity and support adaptive personalized training to meet every patient's rehabilitation needs. In this paper, we focus on integrating adaptivity and personalization in rehabilitation training for MS patients. We introduced the automatic adjustment of difficulty levels as an adaptation that can be provided in individual and collaborative rehabilitation training exercises for MS patients. Two user studies have been carried out with nine MS patients to investigate the outcome of this adaptation. The findings showed that adaptive personalized training trajectories have been successfully provided to MS patients according to their individual training progress, which was appreciated by the patients and the therapist. They considered the automatic adjustment of difficulty levels to provide more variety in the training and to minimize the therapists involvement in setting up the training. With regard to social interaction in the collaborative training exercise, we have observed some social behaviors between the patients and their training partner which indicated the development of social interaction during the training.

## 1. Introduction

Multiple Sclerosis (MS) is an autoimmune, chronic, and progressive disease of the central nervous systemof humans. People with MS suffer from damaged nerves which lead to the progressive interference with functions that are controlled by the nervous system such as vision, speech, walking, writing, and memory, causing severe limitations of functioning in daily life. To date, no cure has been found for MS yet. Therefore, for MS patients, the aim of rehabilitation training is different compared to any other disease. Rehabilitation training will not completely recover MS patients; however, it may improve their functional mobility and quality of life.

People who are in need of any kind of rehabilitation are individuals with their own characteristics and needs. Although they might be subjected to the same background cause for rehabilitation, the stage of their condition or the severity of their disease may differ which requires different treatments and forms of rehabilitation. In the case of MS, the individual differences among its patients are quite prominent due to the great variation of MS symptoms and the impact levels of the disease. For example, their physical abilities, which are largely influenced by the degree of muscle weakness experienced by the patients, differ a lot. The difference in physical abilities has an impact on the course of the rehabilitation training. Some training tasks may be difficult for some patients because of their limited capabilities due to their high degree of muscle weakness, while other patients experience less problems in performing those tasks.

During rehabilitation, each patient progresses in different ways; thus, the training exercises must be tailored to each individual differently. For example, the difficulty of an exercise should increase faster for those who are progressing well compared to those who are having trouble performing the exercise. The condition of patients may also change over time: it can deteriorate according to the progress of the disease or it can improve as a result of the treatment and rehabilitation efforts. This change of condition should be taken into account to ensure providing the right level of rehabilitation training to the patient at the right time. Therefore, due to the diversity among MS patients, it would be unwise to offer the same rehabilitation training to every patient. This situation demands a suited, personalized rehabilitation training to meet every patient's needs.

To acquire a good result of rehabilitation, it is necessary to maintain patient motivation. Generally, rehabilitation involves the same training exercises that should be performed repetitively and for a long period of time. Using games as the training platform has been considered to maintain and enhance patients' motivation during rehabilitation, especially collaborative games in which social interaction is incorporated. However, the usage of game-like training exercise is not the ultimate solution. Some patients may feel less motivated when finding the game to be too easy or too difficult or when reaching a certain point in the training where they become bored with the game. Therefore, rehabilitation training should be set at an appropriate level of challenge or difficulty to maintain the motivation of patients. This raises the need of adaptivity in the rehabilitation training to ensure the effectiveness of the rehabilitation.

Our work focuses on the idea of integrating adaptivity and personalization into the rehabilitation training for MS patients and investigating to what extent the adaptive personalized training may contribute to a successful neurological rehabilitation. This paper firstly discusses the survey of related work concerning adaptation in rehabilitation training, followed by a description of our rehabilitation system developed to support personalized rehabilitation training for MS patients. We further elaborate on the investigation of integrating the adaptivity of automatic difficulty level adjustment in the rehabilitation training for MS patients, including the user studies carried out with a group of MS patients to acquire their feedback.

## 2. Related Work

In this section, we first provide a brief overview of research which has studied adaptation in rehabilitation training. We then discuss several works which have investigated the usage of virtual environments as the platform for rehabilitation training. Lastly, we describe a number of studies which combined both and attempted to integrate adaptation in virtual environments for rehabilitation training.

### 2.1. Adaptation in Rehabilitation Training

The integration of adaptation in rehabilitation training has been the focus of several studies [1–4]. These studies have mainly investigated the integration of adaptivity in robot-assisted rehabilitation training which aimed at providing a personalized training to the patients according to their individual characteristics, needs, and abilities. Moreover, the adaptivity is intended to facilitate an automated training system to minimize the therapist's effort in manually adjusting the rehabilitation training.

Jezernik et al. [1, 4] studied the adaptation in rehabilitation training of locomotion for stroke and spinal cord injured patients. An automated treadmill training system was introduced using a robotic rehabilitation device to increase the training duration and reduce the physiotherapists' effort. A clinical study on six spinal cord injured patients showed that the treadmill training with adaptive gait patterns increases the motivation of the patient and gives him/her the feeling that they are controlling the machine rather than the machine controlling them. Kahn et al. [2] described the integration of adaptive assistance into guided force training as part of the upper extremity rehabilitation for chronic stroke patients. An adaptive algorithm was developed to individually tailor the amount of assistance provided in completing the training task. This algorithm has been evaluated with one patient in a two-month training program which showed significant improvements in the patient's arm function reflected by the performance increase of functional activities of daily living such as tucking a shirt and stabilizing a pillow. Kan et al. [3] presented an adaptive upper-limb rehabilitation robotic system for stroke patients which accounts for the specific needs and abilities of different patients. Using the decision theoretic model, the system autonomously facilitates upper-limb reaching rehabilitation by tailoring the exercise parameters and estimating the patient's fatigue based on the observation in his/her compensation or control of movements. The system performance was evaluated by comparing the decisions made by the system with those of a human therapist. Overall, the therapist agreed with the system decisions approximately 65% of the time and also thought the system decisions were believable and could envision this system being used in both a clinical and home setting.

### 2.2. Virtual Environments for Rehabilitation Training

Another emerging technology that has been widely applied in rehabilitation is the virtual environment technology. Virtual environments provide a variety of potential benefits for many aspects of rehabilitation training. Schultheis and Rizzo [5] discussed a number of advantages for the use of virtual environments in rehabilitation. One key benefit is that virtual environments are rather naturalistic or “real-life” environments, which may allow the users or patients to be immersed within the environment and “forget” that they are in a rehabilitation training session. Within virtual environments, patients are also facilitated to perform the rehabilitation training tasks using 3D interaction, which gives a close resemblance to the actual movements in the real world. Schultheis and Rizzo [5] also pointed out that virtual environments facilitate the design of individualized training environments where therapists can better tailor the training exercises based on an individual's abilities and needs, and they can also easily apply gradual increments of difficulty and challenge. Another benefit is that virtual environments allow the introduction of gaming factors into the rehabilitation scenario to enhance motivation of patients.

Holden [6] provided a thorough overview of the use of virtual environments in the field of motor rehabilitation. In the context of motor rehabilitation, several studies have shown that patients with motor impairments are able to train their motor skills in virtual environments and transfer these abilities to the real world. Furthermore, these studies have also indicated that motor learning in a virtual environment may be superior to that in a real environment, for example, a study described in Webster et al. [7] which compared two groups of patients with stroke and unilateral neglect syndrome in a wheelchair training program. The group which had trained in a virtual environment hit significantly fewer obstacles with their wheelchair during the real world obstacle avoidance test than the group which only trained in a real environment. Another study described in Jaffe et al. [8] investigated chronic stroke patients in a training to avoid obstacles during walking. Patients using a virtual environment-based training showed greater improvement in a fast paced velocity test in comparison to the patients who trained in the real world.

In Holden [6], an extensive description of the various virtual environments that have been developed for rehabilitation purposes of patients was provided. This includes rehabilitation for stroke (upper and lower extremity training and spatial and perceptual-motor training), acquired brain injury, Parkinson's disease, orthopedic disorders, vestibular disorders (balance training), wheelchair mobility training, and functional activities of daily living training. A review on these research initiatives showed that a wide variety of clinical applications using virtual environments has been developed and tested. Mainly, the virtual environments consist of scenes that were designed to be simple, therapeutically meaningful tasks to the targeted patients such as making coffee, lifting a cup to the mouth, using an automated teller machine (ATM), and way-finding. Virtual environments have been considered not as a treatment for motor rehabilitation in itself but more as a new technological tool that can be exploited to enhance motor rehabilitation training.

Over the past few years, there is an increasing research interest in the development of virtual environments for use in stroke rehabilitation. Virtual environments are considered beneficial in stroke rehabilitation because they enable more precisely controlled training settings, intensive practice with easier repetition of tasks, automatic recording of training progress, and more enjoyable and compelling interaction for the patients. Some researchers developed virtual environments based on the literal translation of activities of daily living such as self-feeding, bathing, and dressing or grooming. For example, Edmans et al. [9] developed a virtual environment to train a self-feeding task (i.e., making a hot drink). An evaluation study with 50 stroke patients showed that their performance of hot drink-making in the real world and in the virtual environment were correlated, which may indicate the usefulness of such virtual environment as a rehabilitation tool for stroke patients.

Other researchers chose to enclose the rehabilitation tasks in game-like exercises, with the purpose of adding fun and challenges into rehabilitation training to increase or maintain the patient's motivation. For instance, Alankus et al. [10] developed a series of home-based stroke rehabilitation games which make use of inexpensive devices feasible for home use such as Wii remotes and webcams. Two examples of the games are the helicopter game in which the patients have to maneuver a flying helicopter using their arm to avoid hitting buildings and to collect fuel cells in the air and the baseball catch game in which the patients have to differentiate balls and control the baseball glove to catch baseballs and avoid basketballs. The preliminary evaluation with one therapist and four stroke patients showed that the games motivated the patients and they used the right motions required for the rehabilitation training. Saposnik et al. [11] described the first randomized clinical trial on the effectiveness of virtual reality using the Wii gaming system, namely, VRWii, on the arm motor improvement in stroke rehabilitation. The results showed that the VRWii gaming technology represented a feasible, safe, and potentially effective alternative to facilitate rehabilitation therapy and enhance motor function recovery in stroke patients.

### 2.3. Adaptation in Virtual Environments for Rehabilitation Training

As previously discussed in Section 2.1, it is essential to provide a personalized rehabilitation training which is suited according to the individual characteristics of patients. Patients involved in rehabilitation have a wide range of needs and abilities which may benefit from integrating adaptivity into their rehabilitation training. Adaptivity enables offering a tailored training with minimal efforts from the human therapists as it decreases the dependence on them to continuously monitor the patient's progress and manually adjust the training program.

Several studies mentioned in Section 2.2 have acknowledged the potential of developing virtual environment applications for the purpose of rehabilitation training. These applications may also benefit from adaptivity since it allows to dynamically adjust the parameters of the virtual environment as the training tool to provide a suited, personalized training to every patient based on his/her current needs and abilities. It may also be necessary to integrate adaptivity in virtual environments for rehabilitation training due to the fact that the complex 3D interaction may introduce extra difficulties and eventually influence patients' performance during the training sessions. Adaptivity may reduce this effect by adjusting the virtual environment according to the patient's level of interaction.

This section discusses several previous studies which highlighted the integration of adaptation in virtual environments that were developed for the purpose of rehabilitation training. Ma et al. [12] stated that virtual reality systems for rehabilitation can benefit a group of patients with a great diversity through adaptation. In their study, several adaptive virtual reality games for rehabilitation of stroke patients with upper limb motor disorders have been developed. The information of patient performance is used to enable automatic progression between difficulty levels in the games. The elements of the games are designed to be adaptive and to change dynamically according to how well or badly the patient is performing. An initial evaluation from patients showed positive feedback since they enjoyed training while playing the game and they felt more motivated.

Cameirão et al. [13] have developed an adaptive virtual reality based gaming system for the upper extremities rehabilitation of acute stroke patients. They proposed a multitask adaptive rehabilitation system that provides a task-oriented training with graded complexity. The basic training consists of a virtual reality game where flying spheres move towards the patient. These objects have to be intercepted using the virtual arms which are controlled by the patient's arm movements. As the first task, the patients have to perform the hitting task to train range of movement and speed. The second task is the grasping task to train finger flexure and then finally the placing task to train grasp and release is the last task. Based on the individual performance of the patient during training, the difficulty of the task is adapted by modulating several parameters such as the speed of the spheres, the interval of appearance between consecutive spheres, and the range of dispersion in the field. The impact of the system on the recovery of patients was evaluated with 14 patients. The results suggested that the system induces a sustained improvement during therapy with observed benefits in the performance of activities of daily living.

Barzilay and Wolf [14] developed an online biofeedback-based adaptive virtual training system for neuromuscular rehabilitation. The system employs an artificial intelligence learning system that learns from real-time biofeedback and produces online new patient-specific virtual physiotherapy missions. In the training, the patient is asked to follow the virtual tasks (i.e., trajectories) displayed to him/her by making upper limb movements accordingly. The performance is then modeled by tracking and recording their kinematics and muscle activity (measured through electromyography (EMG) signals) using a motion tracking system. Based on this trained model, the system changes and adapts the task displayed to the patient which results in a new virtual trajectory as the training exercise. This adaptive loop of the system is continuously repeated to provide a real-time adaptive rehabilitation virtual training system for better neuromuscular rehabilitation.

According to Holden [6], the use of mixed reality, which combines physical and virtual environments, in rehabilitation training can provide adaptive scenes for interactive practice and feedback that engage the patient physically and mentally. Duff et al. [15] presented an adaptive mixed reality rehabilitation training system to help improve the reaching movements of patients who suffer hemiparesis from stroke. The system provides real-time, multimodal, customizable, and adaptive feedback based on the movement patterns of the patient's affected arm and torso during the reaching movement. The kinematic data is used to assess the movement and adapt the parameters linked to the feedback presentation and the physical environment that determines the type of task. The feedback is provided via innovative visual and musical forms that present a stimulating, enriched environment for the patient's training. For example, the audio feedback in the form of music is intended to encourage patients to perform the desired reaching movements. The velocity of the patient's hand controls the rhythm of the music; when the patient is moving too slow, the music is played with a slower rhythm and when the patient moves with nonsmooth acceleration or deceleration, there will be abrupt changes in the pace of the music. Additionally, the sequence and intensity of tasks can also be adapted to better address each patient's rehabilitation needs. After training with the interactive mixed reality system, three chronic stroke patients showed improved reaching movements. Integrating mixed reality environments into the training was argued to promote an easier bridging in motor learning between the virtual and physical worlds. In one of their following research, Baran et al. [16] presented the design of a home-based adaptive mixed reality system for stroke rehabilitation. The system, which consists of a custom table, chair, and media center, is designed to be easily integrated into any home to provide an engaging long-term reaching task therapy for stroke patients.

### 2.4. Summary of Related Work

In this section, we have discussed several research efforts, which have mainly investigated the integration of adaptivity in robot-assisted rehabilitation training. Besides providing a personalized training, the adaptivity integrated in the studies was intended to minimize the dependence from the therapists for manually adjusting the rehabilitation training. Virtual environment technology has been considered to be beneficial and widely applied in rehabilitation. We also have discussed a number of research initiatives that used virtual environments as part of the rehabilitation training platform and demonstrated the patients' ability to train in virtual environments and transfer the trained skills to the real world. Furthermore, we have described several studies which have attempted to integrate adaptivity in the virtual environment training system for achieving better results and patient's engagement in rehabilitation. Mostly, the adaptivity takes form in automatically adjusting the parameters of the virtual environment to alter the difficulty levels in the rehabilitation exercises based on the patient's current training performance and training progress.

We observed that previous studies mainly focused on robot-assisted rehabilitation training for stroke patients. In our work, we aim to develop a haptic-based rehabilitation system (combining robot-assisted rehabilitation and virtual environments technologies) to support a systematic and personalized upper limb rehabilitation training for MS patients.

## 3. Rehabilitation for Multiple Sclerosis

Rehabilitation for people with MS mainly aims for improving their functional mobility and quality of life, rather than attaining their full recuperation from the disease. This is due to the fact that MS is an incurable illness. In the past, physical training was not advised for MS patients due to the opinion that it would advance the deterioration. Now, however, performing physical training is often part of therapy and rehabilitation efforts for MS patients in parallel with taking medications. A number of studies have shown beneficial effects of physical training in MS regarding lower limb muscle strength, exercise tolerance level, functional mobility (i.e., balance and walking), and quality of life, while no harmful effects were reported [17, 18].

Little research has investigated the therapeutic value of upper limb, in particular arm, rehabilitation for MS patients. Upper limb rehabilitation is considered important since upper extremity dysfunction strongly influences the capacity of MS patients to perform several activities of daily living (ADL) such as self feeding (e.g., eating, filling, or drinking a cup of coffee), bathing, grooming, dressing, and taking medications.

### 3.1. Robot-Assisted Upper Limb Rehabilitation

Keys to a successful neurological rehabilitation are training duration and training intensity [19]. As time dedicated to upper limb training may be limited in a formal training session, additional therapeutic modalities may be necessary to enable MS patients to train independently of the therapist. Robot-assisted rehabilitation and virtual environment technologies have been considered to be promising to provide an effective, independent upper limb rehabilitation training [20, 21]. Robot-assisted rehabilitation allows high-intensity, repetitive, task-specific, and interactive treatment of the impaired upper limb. Virtual environments facilitate patients to perform the training tasks with their upper limb using 3D interaction, which gives a close resemblance to the real movements in the real world.

In the context of a European research project (INTERREG-IV program “Rehabilitation robotics II”), we investigate the effects of robot-assisted upper limb rehabilitation training for MS patients. Our work combined two technologies, robot-assisted rehabilitation and virtual environments, by first investigating the feasibility of using a phantom haptic device for arm rehabilitation in MS patients [22, 23]. Further, a complete haptic-based rehabilitation system,* I-TRAVLE*, was developed to support a systematic and personalized upper limb rehabilitation training for MS patients [24].

**Figure 1:**
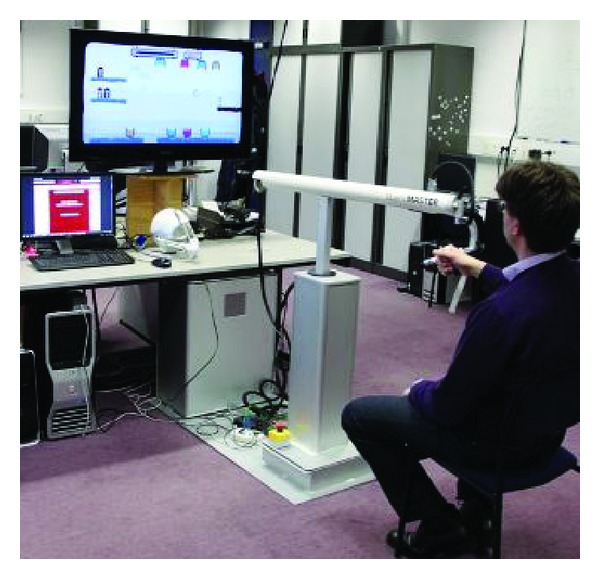
I-TRAVLE system setup.

I-TRAVLE (*Individualized, Technology-supported, and Robot-Assisted Virtual Learning Environments*) consists of a hardware and software system setup as depicted in Figure 1. The main component of the hardware system is a haptic robot, the MOOG HapticMaster, that functions as both input and output devices. As an input device, it allows patients to interact with the software applications that deliver the training exercises. As an output device, it provides haptic feedback during the training by guiding or hindering the patient's arm movement with exerted forces. The HapticMaster is equipped with a peripheral device, the ADL Gimbal, where the patients' hand is placed and secured using the attached brace while performing the training exercises. A large display, a full HD 40′′ Samsung TV screen, is used as a visual display to project the training exercises and is placed behind the HapticMaster approximately 1.5 m in front of the patient. A complete description of the I-TRAVLE hardware system with the adjustments made for the context of MS training can be found in de Weyer et al. [25].

**Figure 2:**
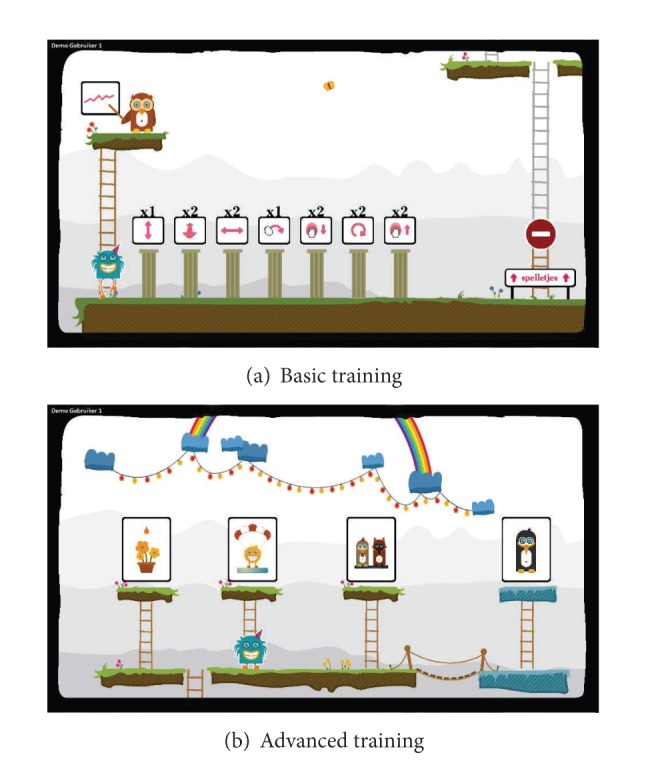
The patient interface.

**Figure 3:**
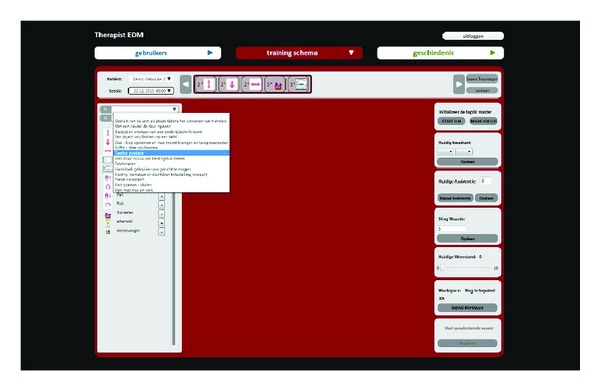
The therapist interface.

The main components of the software system of our I-TRAVLE system are the training exercises, the patient interface, the therapist interface, and the central database. Two types of training exercises were developed, namely, the basic and the advanced training exercises. We will discuss the training exercises in Section 3.2. The patient interface, as shown in Figure 2, is created to enable patients to independently start performing and navigating through the exercises predefined by the therapist.  Figure 2(a) shows how the patient interface looks like in the basic training and Figure 2(b) shows the interface for the advanced training. Another interface was developed for the therapist, as shown in Figure 3, to manage the user data, personalize therapy sessions, and review the data logged from the performed training exercises. The central database stores all the data about the training exercises and patients' performance. A more detailed description of the I-TRAVLE software system can be found in Notelaers et al. [26].

Several other researches have been done in the course of the I-TRAVLE system development regarding the influence of visual aspects on the capabilities of MS patients. People with MS often suffer from visual system disorders and cognitive dysfunctions, which may influence their capabilities while navigating in a virtual environment. Van den Hoogen et al. [27] investigated the impact of visual cues such as shading on navigation tasks in a virtual environment for MS patients. The study showed that the addition of shade below patients' current position in the virtual environment improves speed during the task and reduces the time spent on the task. Van den Hoogen et al. [28] discussed a study to comparatively test the effectiveness of two characteristics of virtual environments, namely, the stereo visualization and the graphic environment, in rehabilitation training when utilizing a 3D haptic interaction device. The experiment results showed that the use of stereoscopy within a virtual training environment for neurorehabilitation of MS patients is most beneficial when the task requires movement in depth. Furthermore, the use of 2.5D in the graphic environment implemented showed the highest efficiency and accuracy in terms of patients' movements.

### 3.2. Individual Training Exercises for Upper Limb Rehabilitation

To keep up the motivation of patients and strive for a successful rehabilitation trajectory, it is essential to give them training exercises that are meaningful in supporting their functional recovery [29]. The development of the I-TRAVLE training approach was inspired by the T-TOAT (technology-supported task-oriented arm training) method of Timmermans et al. [30]. T-TOAT is a technology supported (but not haptic) training which allows integration of daily tasks. Similar to the T-TOAT method, I-TRAVLE divides an activity of daily living (ADL) into skill components and trains the skill components, first every component separately and later several components combined.

**Figure 4:**
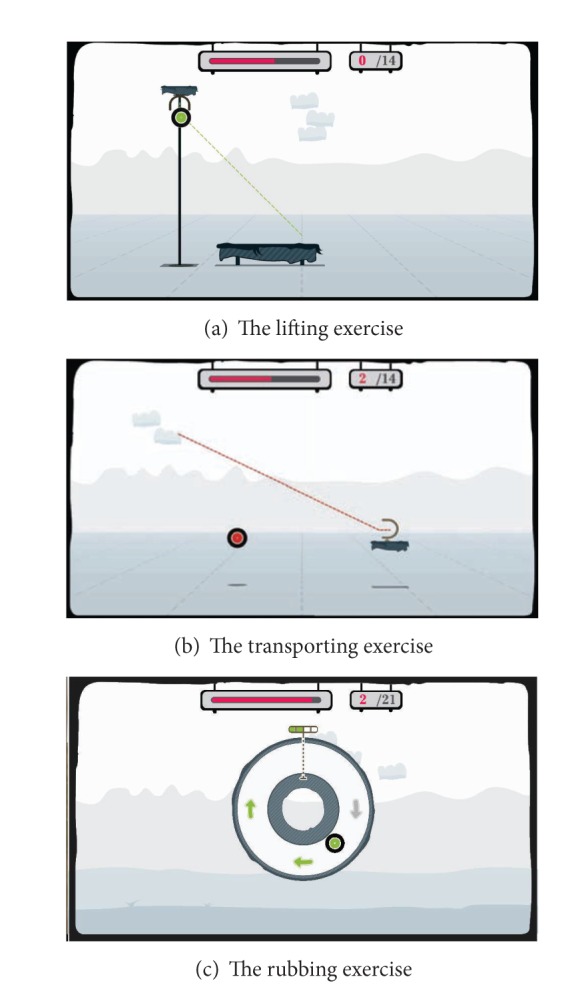
The basic training exercises for upper limb rehabilitation.

The I-TRAVLE training exercises were designed based on the skill components that patients need to train related to their upper limb rehabilitation. Two types of training exercises are provided, namely, basic training exercises which include only one skill component and also advanced training exercises which combine multiple skill components. Seven basic training exercises were developed in I-TRAVLE as follows: pushing, pulling, reaching, turning, lifting, transporting, and rubbing.  Figure 4 shows three examples of the basic training exercises: lifting (Figure 4(a)), transporting (Figure 4(b)), and rubbing (Figure 4(c)).

**Figure 5:**
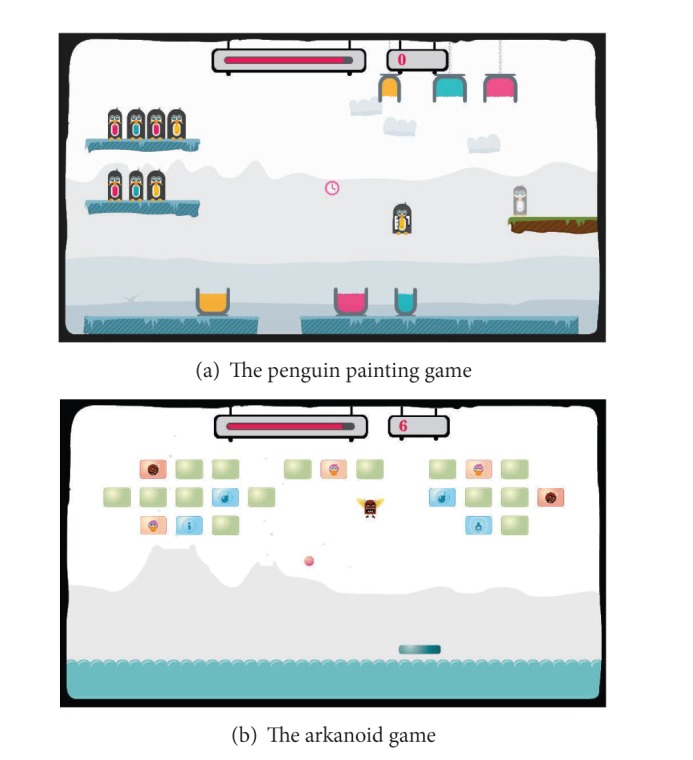
The advanced training exercises for upper limb rehabilitation.

In the advanced training exercises, several skill components were combined into a game-like training exercise as illustrated in Figure 5. Until now, four advanced training exercises have been designed as follows: penguin painting (Figure 5(a)), arkanoid (Figure 5(b)), egg catching, and flower watering. Normally in a therapy session, a patient first trains with several basic training exercises. Depending on the progress of the patient, the therapy session can be continued with the games (i.e., advanced training exercises).

**Figure 6:**
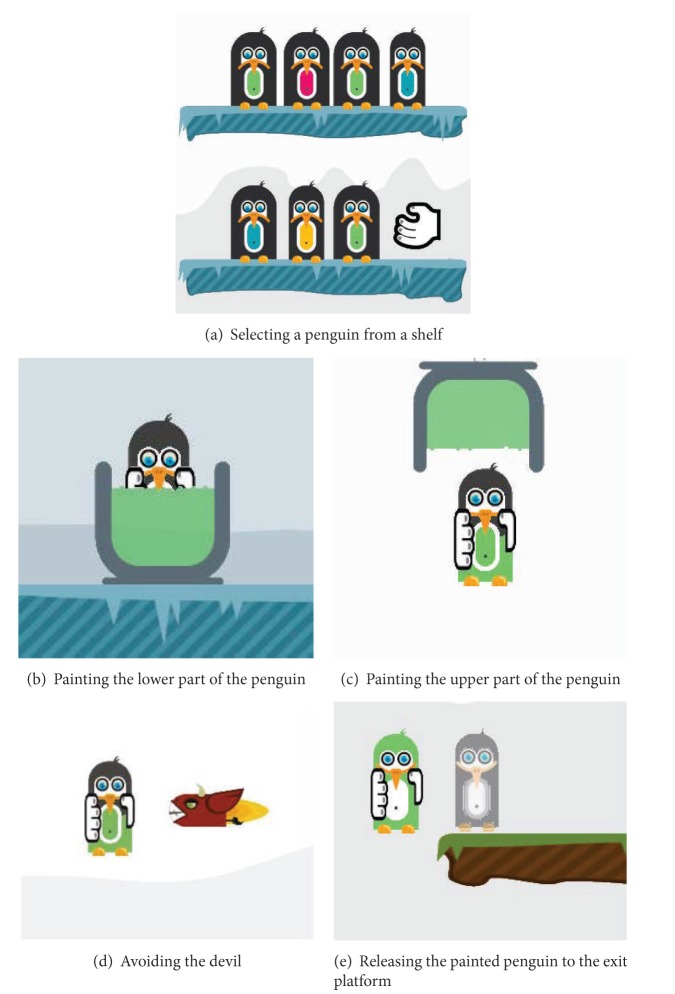
Illustration of the penguin painting game.

One example of the advanced or game-like training exercises is the penguin painting game as illustrated in Figure 5(a). This game was designed based on two skill components: lifting and transporting. Lifting is one of the important skill components in the upper limb rehabilitation for MS patients. In the penguin painting game, the patient has to collect as many points as possible within a certain time period by painting as many penguins as possible with the right color.  Figure 6 illustrates how the penguin painting game is played. On the left side, there are two shelves with penguins waiting to be painted. The patient has to select one penguin from a shelf (Figure 6(a)) and paint it according to the color of its belly. To paint, the patient needs to bring the penguin to the corresponding buckets by dipping it into the bottom to paint the lower part of the penguin (Figure 6(b)) and continue dipping it into the top bucket to paint the upper part of the penguin (Figure 6(c)). While painting, the patient must hold the penguin long enough to effectively apply the color and train for stabilization in this way. At some points during the game, a devil that tries to capture the penguin appears (Figure 6(d)) and must be avoided in order to not lose the penguin already in hand. Every time the patient finishes painting a penguin, the colored penguin must be transported to the exit platform on the right side (Figure 6(e)).

The aforementioned training exercises discussed in this section are designed to be part of individual rehabilitation training for MS patients. This means that the patient will perform the training exercises on his/her own. In this case, the role of the therapist is to determine which training exercises the patient has to perform and also to personalize the therapy sessions according to the patient's current condition and needs. In another case, the therapist will extend his/her role to not only manage the therapy sessions but also take an active part in the therapy sessions by collaborating with the patient in performing the training exercises. This so-called collaborative rehabilitation training will be further discussed in the next section.

### 3.3. Collaborative Training Exercises for Social Rehabilitation Program

Typically exercises in robot-assisted rehabilitation involve a patient training on his/her own (with a therapist's supervision) and performing repetitive movements for a certain time period predetermined by the therapist. Even when gaming elements are integrated in the training exercises, it is crucial to exploit a variety of motivational techniques to avoid bored patients and patients that are stopping the therapy due to the high intensity and repetitiveness of the exercises. Therefore, we explore different motivational aspects to increase the patient's engagement in the therapy.

Social support has been demonstrated to be beneficial for the engagement and motivation of patients in a rehabilitation context. Van den Hoogen et al. [32] have performed a study on rehabilitation needs of stroke patients, which indicated that social support is critical for patient motivation in order to adhere to the necessary regime of rehabilitation exercises in the chronic phase of stroke. In addition to using games, patient's motivation during rehabilitation can be maintained and further enhanced through the incorporation of social interaction into the training exercises offered in the rehabilitation program.

During rehabilitation, patients can receive social support from different groups in their social network not only from their family members and friends but also from other support groups such as their therapists, caregivers, or fellow patients. These people can extend their form of social support to the patients by actively participating in the therapy sessions. This support can be manifested in two different forms:* sympathy* and* empathy* [33, 34]. Both depend on the types of relationships built based on shared emotions and sense of understanding between the persons. Two possible social scenarios can exist in this context.

(1) *Sympathetic*. This social situation occurs when a person shows the ability to understand and to support the condition or experience of the patient with compassion and sensitivity. For example, healthy family members and close friends may be able to show sympathy towards MS patients.
 One can imagine a situation, where a patient is visited by a family member (e.g., a daughter). This family member could show her sympathetic support through her help in the rehabilitation program by performing the collaborative training exercise together with the patient.
(2) *Empathetic*. This social situation happens when a person shows the ability to coexperience and relate to the thoughts, emotions, or experience of the patient without them being directly communicated. Other fellow patients can easily have empathy towards MS patients due to the resemblance of their condition.
 One can imagine a situation, where two patients are supporting each other by training together at the same time through the collaborative exercise, which makes the training more pleasant and fun.

Social interaction can be incorporated in the rehabilitation training by providing a social play medium which requires a patient to collaborate with his/her supporting partner in performing the training exercises. Thus, the idea is that the patient will keep being engaged and stay motivated in training by collaborating with other people as a training partner. By this, we extend the individual rehabilitation training to a collaborative rehabilitation training, where the training exercises involve collaboration and participation of more than only a single MS patient.

**Figure 7:**
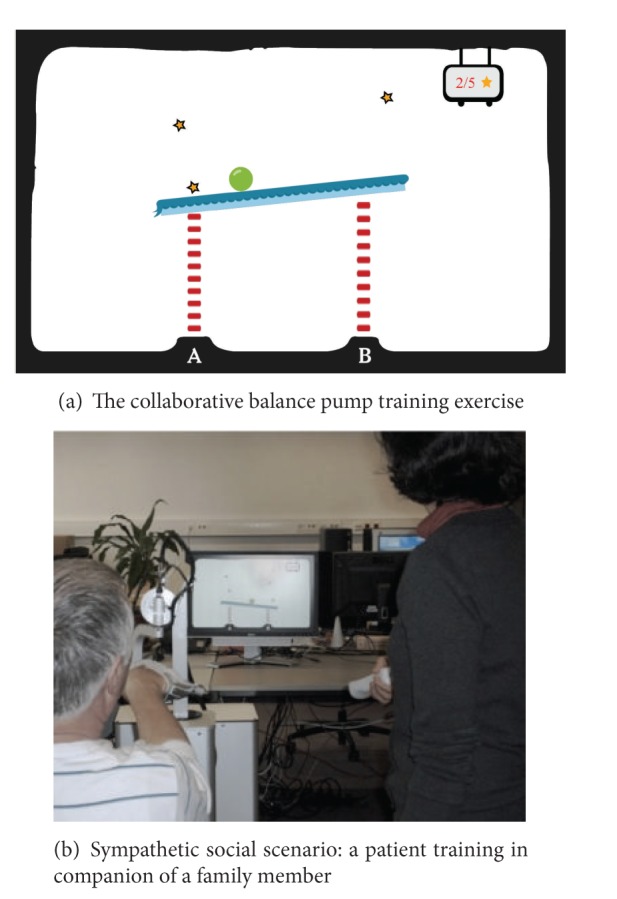
Example of a collaborative training exercise for MS patients. Vanacken et al. [31]

Vanacken et al. [31] have explored the possibility of social rehabilitation training by designing a simple collaborative game-like training exercise, as shown in Figure 7. A collaborative balance pump game was created (Figure 7(a)), where two people “play” together during the therapy session. The goal of the game is to collect all stars, which represent points, by hitting them with a ball rolling over a beam. Both players have to collaboratively control and balance the height of both sides of the beam by pumping each side of it in turns.  Figure 7(b) illustrates the setup of the social rehabilitation training session in a sympathetic scenario, where a patient plays the collaborative balance pump game with a family member. To produce the pumping gesture, the patient uses a HapticMaster and the healthy person uses a WiiMote as input devices.

**Figure 8:**
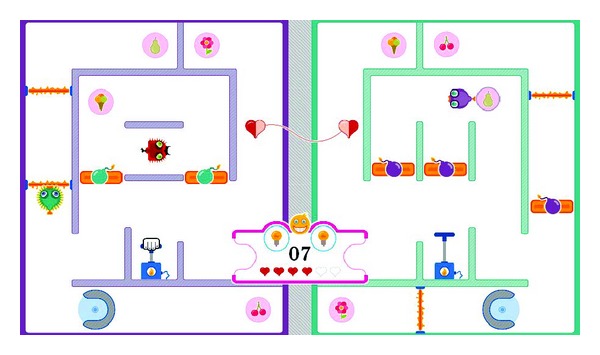
The social maze game.

An informal user study has revealed that most patients and therapists liked and enjoyed training with the collaborative balance pump game described in Vanacken et al. [31]. Inspired by this, we developed another collaborative training exercise,* social maze*, which has more game elements and variations [35]. This collaborative training game was designed based on several skill components (see Section 3.2) that MS patients need to train related to their upper limb rehabilitation: lifting, transporting, turning, pushing, and reaching.  Figure 8 depicts our social maze with all game elements. The goal of this game is to collect all symbols, which represent points, by picking up each symbol and bringing it to the collecting bin. The elements of the game were purposively designed so that two players (i.e., a patient and his/her training partner) have to collaborate as such to achieve the goal. Without collaboration, it is impossible to finish the game.

**Figure 9:**
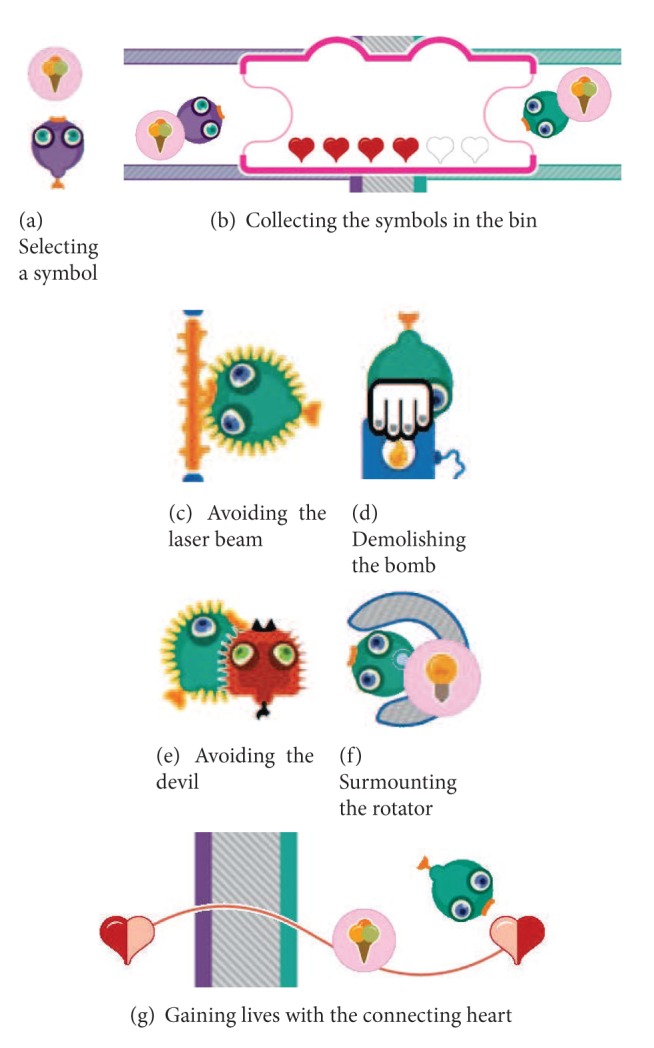
Illustration of the social maze game.

As can be seen in Figure 8, the game area is divided into two. In each part, the player, represented by a fish-like avatar, can move around his/her own maze to collect the symbols.  Figure 9 illustrates how the social maze game is performed. First, the player has to select a symbol (Figure 9(a)). Once the players pick up a symbol, they can place it in the collecting bin (Figure 9(b)) to earn points. Along the way, there are some obstacles such as the laser beams that need to be avoided (Figure 9(c)), the bombs that need to be demolished (Figure 9(d)), the devil that may not be encountered (Figure 9(e)), and the rotators that need to be surmounted (Figure 9(f)). Demolishing the bombs demands a tight collaboration between the players, where one player has to push the bomb trigger (lifting skill component) in order to destroy the bomb blocking the way of the other player. To pass the rotator, the players must enter it and perform the turning movement (turning skill component) to rotate it. When the players are hit by the laser beam or the devil, they will lose a life represented by a heart. To gain more lives, the players must attain the connecting heart (Figure 9(g)).

### 3.4. Personalization in I-TRAVLE

The diversity of MS patients raises the need for personalization in their rehabilitation training. The differences in physical abilities, MS symptoms, and disease progression bring an influence to the patients' condition and their rehabilitation needs. Therefore, offering the same training trajectory to MS patients is a straightforward, yet unwise, approach. A personalized training that complies to each patient's characteristics becomes a necessity to accommodate the patient diversity and ensure an effective and satisfactory rehabilitation. The I-TRAVLE system has been developed with a concern for supporting personalization in upper limb rehabilitation training for MS patients. An exploratory study on the personalization aspects has been discussed in Notelaers et al. [36]. The first aspect considered in the personalization infrastructure of I-TRAVLE was the workspace determination of patients. Due to the difference in patients' abilities in using their upper limb, some patients may have a smaller range of motion than the others. Based on the measurement of their active range of motion (AROM), the setup of the training program can be adjusted accordingly to ensure that the effort required to perform the training exercises is within the capability of the patient and no impossible or harmful arm movements were necessary.

Within I-TRAVLE, normally the therapist manually determines the training program and its exercise parameters in the therapist interface (see Figure 3) to present suitably challenging, individualized rehabilitation training. The therapist can set up a training program according to the patient's desire to train certain activities of daily living. For example, when the patient prefers to train his/her ability of drinking a cup of coffee (i.e., self feeding), the therapist can choose the suitable skill components such as reaching, lifting, and transporting. Providing customizable parameters of these skill components ensures personalization to better suit the patients' capabilities. A personalized training program with the appropriate difficulty level according to the patients' abilities can be established. For instance, to achieve the training of more fine-grained arm movements, the therapist can customize the parameters of the lifting exercise to a higher difficulty. Several kinds of parameters are adjustable, for instance, related to visual or haptic feedback. The amount of haptic feedback that is given when the patient has to follow a certain path in the virtual world influences how easy or how difficult it is for the patient to stay on the path. Figure 4(a) shows the lifting exercise. The green line shows the ideal path the patient has to follow to bring the green disc to the target through the HapticMaster. When the patient deviates too much from the ideal path, the line and disc become orange and finally red (as shown for the transporting exercise in Figure 4(b) to encourage the patient to correct her path. Another example of feedback that is parameterized is illustrated for the skill component rubbing in Figure 4(c). The patient has to move the disc between the two walls of the tube. Haptic feedback makes the inside of the walls either smooth or course grained, so that it is either easy or hard for the patient to slide the disc next to the walls.

Not only the parameters of the skill components can be customized but also personalization can take place in the gaming level. Figure 5(a) shows the penguin painting game. To make the lifting easier, the weight of the penguins can be adjusted. In the arkanoid game (Figure 5(b)), the viscosity of the bar pushing back the ball is also an adjustable haptic parameter.

However, the customization of exercise parameters is dependent on the therapist's judgement and requires the therapist to monitor the difficulty levels and preset parameters during the training session and to intervene to adjust if needed. There might be times when the therapist is not aware of the need for adjusting the training program according to the patient's progress or when the therapist finds a limited time to set up a new training program customized to the patient's current ability. This dependency can be minimized without a conscious effort from the therapist through the integration of adaptivity. Adaptivity enables the system to automatically adjust itself in such a way to support the different context of each patient's rehabilitation training. Tailoring the right training challenge in the right time without any interference from the therapist can be provided by automatically adjusting the difficulty level of the training exercises according to the patient's current training performance [37].

## 4. Towards Adaptive Personalized Rehabilitation Training:* Automatic Difficulty Level Adjustment*

The focus in this work is how we can enhance our personalization strategy by integrating adaptivity into the rehabilitation training, both in individual and collaborative training exercises, for MS patients. Due to the diversity of MS patients, every patient has a different physical ability and condition which may influence the course of their training. For example, one patient might find it very difficult to perform a specific training task, while the other finds it quite easy to perform it. We also have to take into account that every patient progresses differently. It could be the case that one patient may progress slower than the other in performing the rehabilitation training tasks. With adaptation, we aim to keep the patients continue on training at their ease and ensure no major barriers inhibit their interaction during training. For that purpose, we propose the adaptivity of automatically adjusting the difficulty level of the training exercise to be investigated in this study.

**Figure 10:**
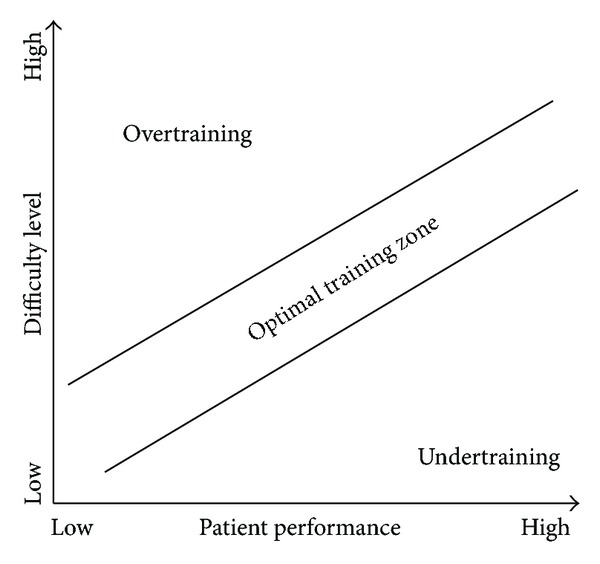
Balancing the difficulty level and patient performance.

To achieve an optimal training experience for MS patients, we were inspired by the flow theory of Csikszentmihalyi [38] to keep the balance between difficulty level and patient performance. As illustrated in Figure 10, we would like to make sure that a patient stays within the “optimal training zone,” where the difficulty level of exercise given to a patient is balanced with his current performance. In the optimal zone, the patient will not experience overtraining or undertraining [39]. Overtraining happens when the patient is asked to perform the exercises with a high difficulty level while his/her performance is still low; thus, the patient is most likely to find the training too difficult and may not be able to perform the training. On the other hand, undertraining happens when a low difficulty level is given to a patient who has a high performance which makes the training not that challenging anymore.

As an optimization strategy to achieve such personalized training, we propose providing the ability to automatically and dynamically adjust the difficulty of the exercise to avoid boredom, provide suitable challenge, and minimize the therapist's involvement as well. This can be done by creating automatic difficulty adjustments according to the patient's performance and progress in the exercise. For this purpose, we need to establish a user model by capturing the patient's performance metrics (e.g., task completion times, scores, and errors) during the exercises and making use of that information to infer the short-term training progress of the patient. This can be considered as a sort of performance-evaluation mechanism. Once established, we can put the user model into practice by applying it to enable adapting the difficulty level whenever necessary. Based on the information from the user model, we can adjust the difficulty of the exercise by making it harder or easier.

In this study, we investigate the possibility of providing automatic difficulty level adjustment in two training exercises of our I-TRAVLE system, namely, the penguin painting game and the social maze game, through two user studies that we have carried out with several MS patients.

## 5. Adaptive Difficulty in Individual Training:* The Penguin Painting Game*

**Figure 11:**
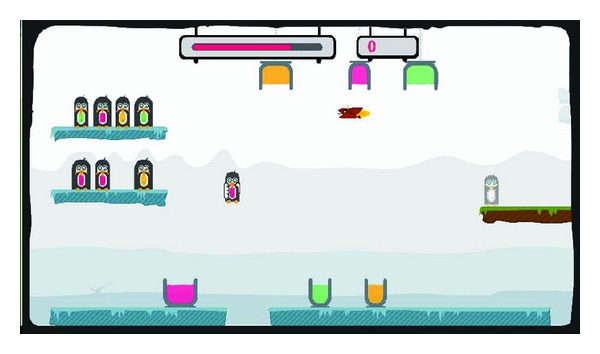
The penguin painting game: initial level.

The penguin painting game is an individual training exercise that was designed as part of the upper limb rehabilitation for MS patients and focuses on training the skill components of lifting and transporting. The goal of this game is to paint penguins with the right color as many as possible within a certain time period (see Section 3.2). At the beginning of the training, every patient starts from an initial level as shown in Figure 11. After two consecutive training sessions, the training progress of the patient is determined based on his/her performance in each session.

If no significant difference of the performance is shown between the training sessions, it is considered that the patient is training on an appropriate level and adaptation will not be triggered. If the patient shows a decrease in his/her performance between the sessions, a lower difficulty level will be automatically offered to the patient in the next session. On the other hand, if an increase of the patient's performance is shown between the training sessions, the system will automatically provide a level with a higher difficulty in the next session.

We define seven different difficulty levels by altering the following game parameters accordingly (see Table 1):

- *size of penguin*: how big the penguins are (small/large);
- *speed of devil*: how fast the devil moves (slow/medium/fast);
- *frequency of devil*: how frequent the devil appears (infrequent/normal/frequent);
- *length of stabilization*: the time required to hold the penguin still (short/normal/long);
- *obstacle wall*: addition of an obstacle wall along the way (no/yes);
- *amount of coloring buckets*: how many coloring buckets exist (2/3/4);
- *width of coloring bucket*: how big the coloring buckets are (narrow/wide);
- *exit platform*: addition of another exit platform which requires patients to place the colored penguins to the size-corresponding platforms (no/yes).

**Figure 12:**
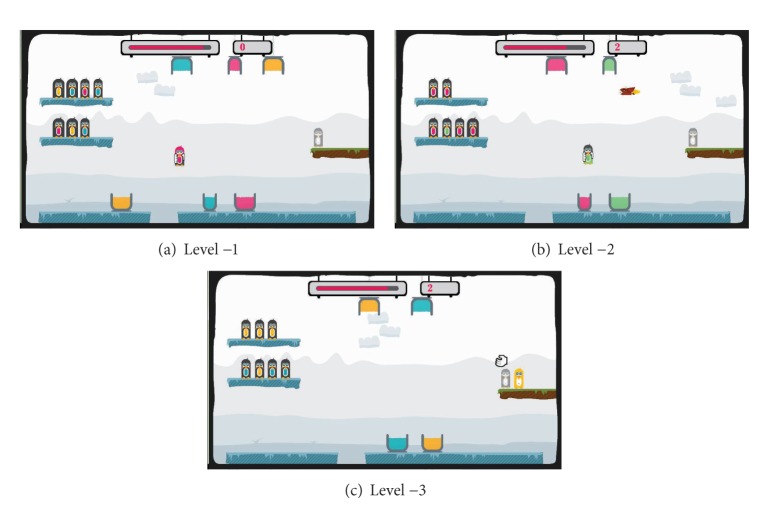
The easy levels in the penguin painting game.

**Figure 13:**
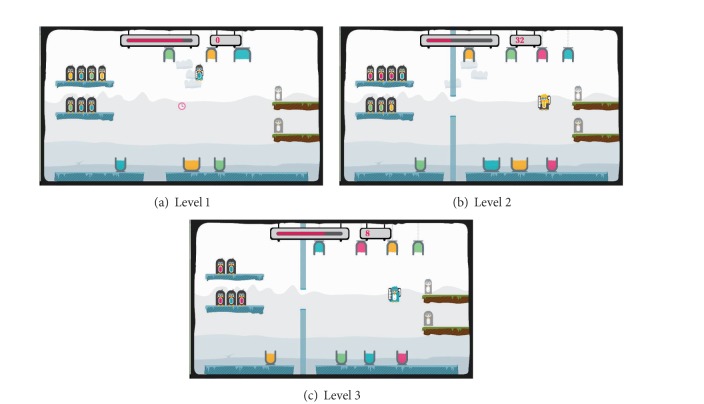
The difficult levels in the penguin painting game.

**Figure 14:**
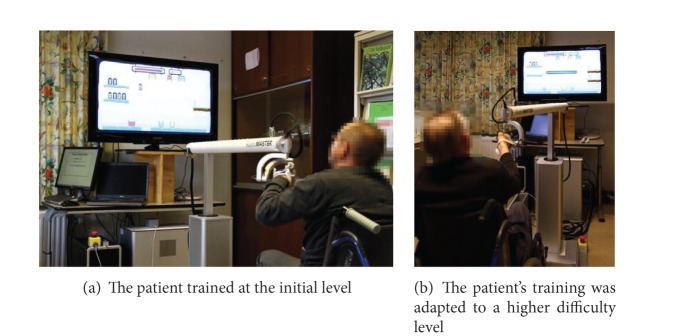
The setup of user study: automatic difficulty level adjustment in the penguin painting game (e.g., 2 exit platforms in the difficult level).

As shown in Figure 12, three different levels are designed as the easy levels in the penguin painting game. Level −1 (Figure 12(a)) is easier than the initial level. Then, one level easier is Level −2 (Figure 12(b)) with Level −3 (Figure 12(c)) being the easiest level.  Figure 14 illustrates the three difficult levels in the penguin painting game. Level 1 (Figure 13(a)) is more difficult than the initial level. Then, one level higher is Level 2 (Figure 13(b)) with Level 3 (Figure 13(c)) being the most difficult level.

To determine the patient's training progress, it is necessary to obtain information of his/her training performance and make a comparison over the last two training sessions. The patient's performance of each training session is calculated as a function of the following performance metrics.

1. * Task Completion Time*.
2. How much time does the patient take to complete one task (i.e., select and transport a penguin)?
3. How much is the slope of task completion times in one training session?
4. * Score*.
5. What score does the patient achieve in one game session?
6. * Error*.
7. How many times does the patient make errors (i.e., hitting the devil, painting with the wrong color)?
8. * Pause*.
9. How many times does the patient make pause actions (i.e., motionless period between steps for longer than 2 seconds)?
10. *Distance*.
11. What is the distance traveled by the patient to complete one task (i.e., select and transport a penguin)?
12. How much is the slope of the distance traveled in one training session?

### 5.1. User Study 1: Automatic Adjustment of Difficulty Level in the Penguin Painting Game

We have integrated the adaptation of automatic adjustment of difficulty levels in the penguin painting game. This results in seven difficulty levels which differ in the exercise parameters as described in Section 5 (see Table 1). We expect that supporting adaptivity in the adjustment of difficulty levels of the training exercises will not only deliver a personalized training to each MS patient but also provide suitable challenge, enable less boredom, and minimize the therapist's involvement. Therefore, we carried out a user study to investigate the outcome of integrating adaptive difficulty into the penguin painting game.

#### 5.1.1. Participants

For the user studies in this research, we recruited a group of participants which consists of nine patients of the Rehabilitation and MS Centre in Overpelt (Belgium) who all suffer from upper limb dysfunction due to MS and were willing to participate in both user studies. The patients were 6 males and 3 females with an average age of 60 years, ranging from 47 to 72 years old. The duration since the MS diagnosis varies between 3 and 34 years, with an average of 20.5 years. Six of them used the left hand to operate the HapticMaster in training, while the other three used their right hand.  Table 2 shows the personal information of each MS patient participating in this research. To have an overview of the severity of their upper limb dysfunction, we obtained their clinical measures as shown in Table 3: upper limb strength (motricity index [40]), upper limb functional capacity (action research arm test [41]), and arm motor function scores (Brunnstrom Fugl-Meyer proximal and distal [42]).

In this first user study, only 8 patients of the group participated. Patient 9 was unable to participate due to his health condition.

#### 5.1.2. Procedure

The user study consisted of seven sessions: two elicitation sessions and five adaptive sessions; all took place on the same day. In the elicitation sessions, participants were asked to perform the penguin painting game in the initial level (see Figures 11 and 14(a)). After two elicitation sessions, the performance metrics of the participant were calculated to determine the progress of his/her training. Based on the information about the training progress over these elicitation sessions, it will be determined for the first adaptive session whether or not the difficulty level should be adapted. Throughout the adaptive sessions, participants were offered an adaptive personalized training in terms of the adjustment of difficulty levels. Three possibilities can happen over the course of five adaptive sessions:* stay at the same level*,* go one level lower*, or* go one level higher* (see Figure 14(b)).

The duration of each session is 3 minutes. After each adaptive session, participants were asked to rate their subjective perception on enjoyment, boredom, challenge, frustration, and fun, on a 5-point scale rating (e.g.,* 1 not at all* to* 5 very much*) based on their experience of performing the adaptive penguin painting game. Averagely, the user study lasted for about 30 minutes per participant.  Figure 14 illustrates the setup of this user study.

#### 5.1.3. Results

**Figure 15:**
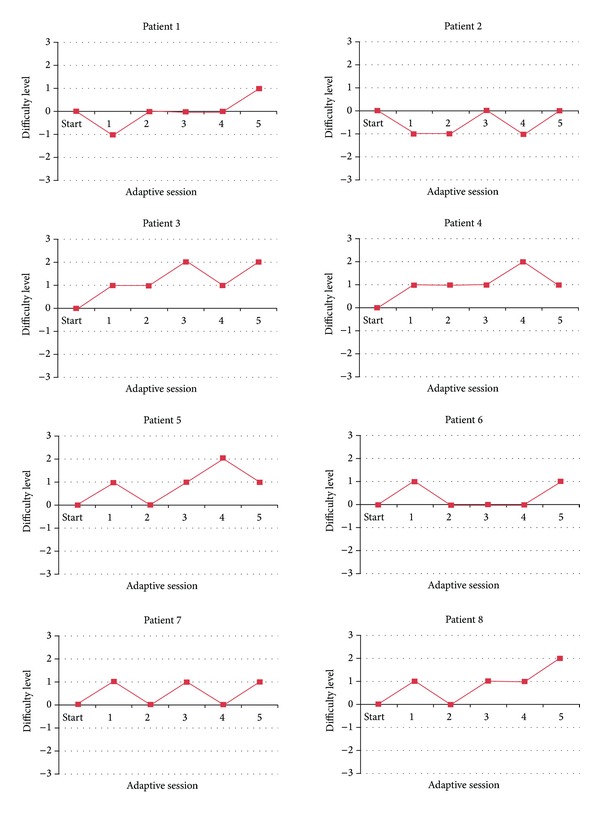
Adaptive personalized training trajectory in the penguin painting game.

We have applied the adaptation of automatic difficulty level adjustment in the penguin painting game, which provided an adaptive personalized training for each participant. Consequently, the training trajectory was different for every participant during the five adaptive sessions. For each session, the participant can experience staying at the same difficulty level, going to a lower level, or going to a higher level, depending on his/her individual performance.  Figure 15 shows the personalized training trajectory for each patient as a result of integrating the adaptive difficulty level adjustment in the penguin painting game. As can be observed, no patient had the same trajectory as the other patient due to the fact that every patient progressed differently. This finding confirms the need for adaptation. Further, we analyzed how adaptation influenced the subjective perception on enjoyment, boredom, challenge, frustration, and fun, across the sessions. Due to the small number of samples and observations in this user study, we used the nonparametric methods for the statistical analysis.

**Figure 16:**
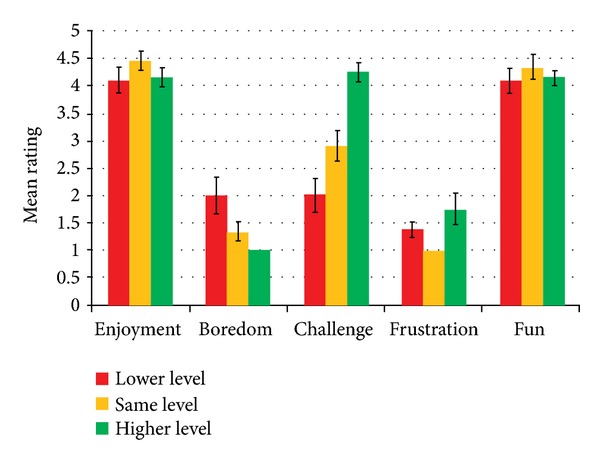
Patient's subjective rating with respect to adaptation.

Based on the patients' subjective responses, we calculated the average ratings of enjoyment, boredom, challenge, frustration, and fun, for the three conditions of adaptation (go to a lower level, stay at the same level, and go to a higher level) as shown by Figure 16. Kruskal-Wallis test showed that no significant differences were found for* Enjoyment*,* Frustration*, and* Fun* between the different conditions of adaptations. This indicates that patients perceived the same level of enjoyment, frustration, and fun eventhough the system introduced an automatic adaptation of difficulty levels in the training exercises. Patients rated a high level of enjoyment and fun (above 4) and a low level of frustration (below 2) in all the conditions.

However, there is a significant difference found for* Boredom* (H(2) = 15.651, *P* < 0.001; 2 for condition 1, 1.33 for condition 2, and 1 for condition 3) and* Challenge* (H(2) = 24.376, *P* < 0.001; 2 for condition 1, 2.89 for condition 2, and 4.25 for condition 3). Mann-Whitney pairwise comparison tests showed that patients felt significantly less bored and more challenged when the training was adapted to a higher level compared to when they had to adapt to a lower level or stayed at the same level (*P* < 0.001).

Furthermore, we observed that some patients have noticed the automatic adaptation to be related to their training progress and they liked the diversity of difficulty levels. We did not inform about the implemented adaptation to the patients before or during the user study. A couple of therapists appreciated the automatic adaptation as it provided the patients with more variety in the training and also gave them more freedom to train on their own without any interference from the therapist to manually adjust the exercise parameters. This kind of adaptation could be useful to determine an appropriate level to start training on a certain day according to the patients condition on that day, thus less determined by the previous training or the therapist.

## 6. Adaptive Difficulty in Collaborative Training: The Social Maze Game

**Figure 17:**
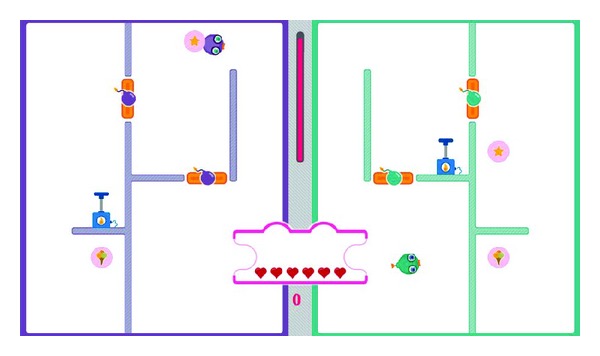
The social maze game: initial level.

The social maze game is a collaborative training exercise that was designed to train several skill components such as lifting, transporting, turning, pushing, and reaching. The goal of this game is to collect all symbols by picking up each symbol and bringing it to the collecting bin (see Section 3.3). To achieve this, the patient should pair and closely collaborate with his/her training partner (e.g., a therapist, family member, or fellow patient). At the beginning of the training, every pair starts from an initial level as depicted in Figure 17.

The same performance-evaluation and adaptation mechanism with the penguin painting game is implemented. First, we obtain an indication of the patient's training progress by comparing the training performance of the last two training sessions. When no training progress is indicated, no adaptation will be triggered and the pair will stay at the same level. When a training progress is indicated, a higher difficulty level will be automatically offered to the pair in the next session. When a training decline is indicated, the system will automatically switch to a lower difficulty level in the next session. It is important to mention here that although collaborative training exercises involve two persons, we mainly focus the adjustment of difficulty level based on the patients' progress in the training since they hold the core function in the rehabilitation. Particularly in the sympathetic scenario, we do not take into account the progress of a healthy person as the training partner since his/her role is solely supporting the rehabilitation training of the patient.

**Figure 18:**
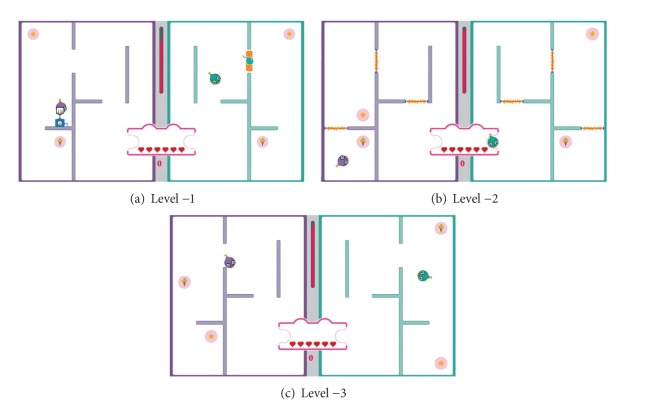
The easy levels in the social maze game.

**Figure 19:**
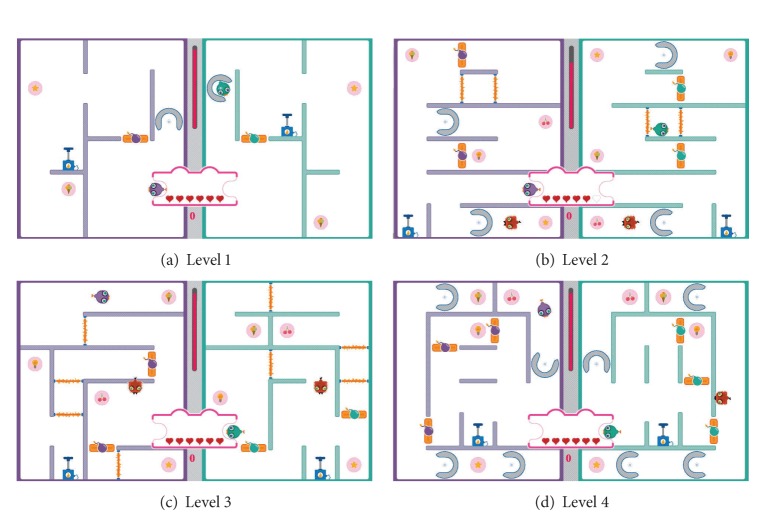
The difficult levels in the social maze game.

We define eight difficulty levels ranging from very easy to very difficult. As shown in Figure 18, three different levels are designed as the easy levels in the social maze game with Level −3 (Figure 18(c)) being the easiest level.  Figure 19 illustrates the four difficult levels in the social maze game with Level 4 (Figure 19(d)) being the most difficult level.

To adjust the difficulty levels, we alter the following game parameters in each level accordingly (see Table 4):

- *viscosity of movement*: how high the viscosity is (very low/low/normal/high/very high);
- *speed of devil*: how fast the devil moves (slow/medium/fast);
- *length of laser beam*: how long the laser beam lasts (short/normal/long);
- *friction of bomb*: the degree of friction that the bomb has (no friction/less friction/much friction);
- *friction of wall*: the degree of friction that the wall has (no friction/less friction/much friction).

In the social maze game, four performance metrics are employed to determine the patient's training progress as follows.

1. * Speed*.
2. How fast can the patient complete one task (i.e., select and transport a symbol)?
3. * Score*.
4. What score does the patient achieve in one game session?
5. * Error*.
6. How many times does the patient make errors (i.e., hitting the devil, hitting the laser beam)?
7. * Pause*.
8. How many times does the patient make pause actions (i.e., motionless period between steps for longer than 2 seconds)?

### 6.1. User Study 2: Automatic Adjustment of Difficulty Level in the Social Maze Game

We have integrated the adaptation of automatic adjustment of difficulty levels in the social maze game. To investigate how patients perceive the outcome of this adaptation, we carried out the second user study with our group of participants as described in Section 5.1.1.

#### 6.1.1. Participants

All nine patients of the group participated in this user study. More information on these participants can be found in Table 2 for their personal information and Table 3 for their clinical characteristics. In this user study, we applied the sympathetic scenario of social rehabilitation training, where a patient collaborates with his/her therapist in performing the social maze game. Only one therapist, whom every patient has a good relationship with, participated in this user study. The Novint Falcon was used as the input device for the therapist. All patients used the HapticMaster as the input device.

#### 6.1.2. Procedure

Similar to the previous study described in Section 5.1.2, the user study consisted of seven sessions: two elicitation sessions and five adaptive sessions. In the elicitation sessions, pairs of participants (i.e., one patient and the therapist) were asked to perform the social maze game in the initial level (see Figure 17). After two elicitation sessions, the performance metrics of the patient were calculated to determine the progress of his/her training. Depending on the observation of the training progress, the system will determine how the difficulty level should be adjusted throughout the adaptive sessions. There can be three possible results of the adaptation:* stay at the same level*,* go one level lower*, or* go one level higher*.

**Figure 20:**
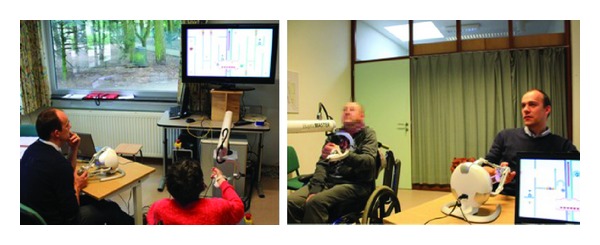
The setup of user study: automatic difficulty level adjustment in the social maze game.

After each adaptive session, patients were asked to rate their subjective perception on difficulty and enjoyment, on a 5-point scale rating (e.g.,* 1 not at all* to* 5 very much*) based on their experience of performing the social maze game. Averagely, the user study lasted for about 30 minutes per participant.  Figure 20 illustrates the setup of this user study.

#### 6.1.3. Results

**Figure 21:**
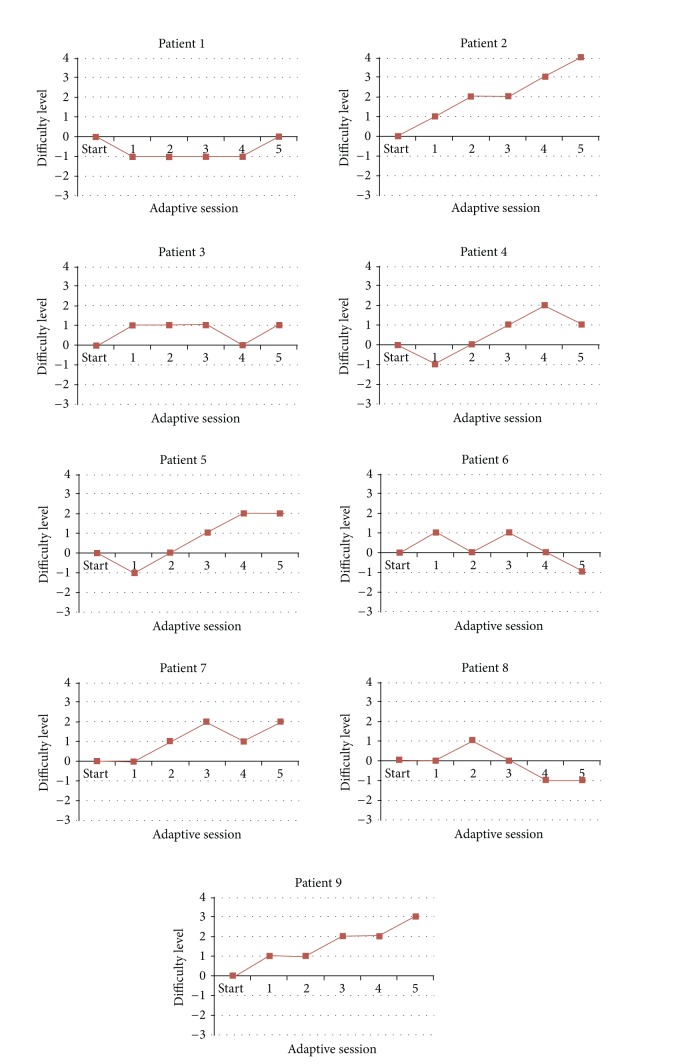
Adaptive personalized training trajectory in the social maze game.

We have applied the adaptation of automatic difficulty level adjustment in the social maze game. Since the adaptation resulted in a personalized training, each patient had a unique training trajectory which differs from each other during the adaptive sessions.  Figure 21 shows the personalized training trajectory for each pair of participants as a result of integrating adaptive difficulty in the social maze game. No pair of participants had exactly the same trajectory as the other, which confirms the need for adaptation based on the patient's individual performance. We analyzed how adaptation influenced the subjective perception on difficulty and enjoyment across the sessions. We used the nonparametric statistics because of the small number of samples and observations in this user study.

**Figure 22:**
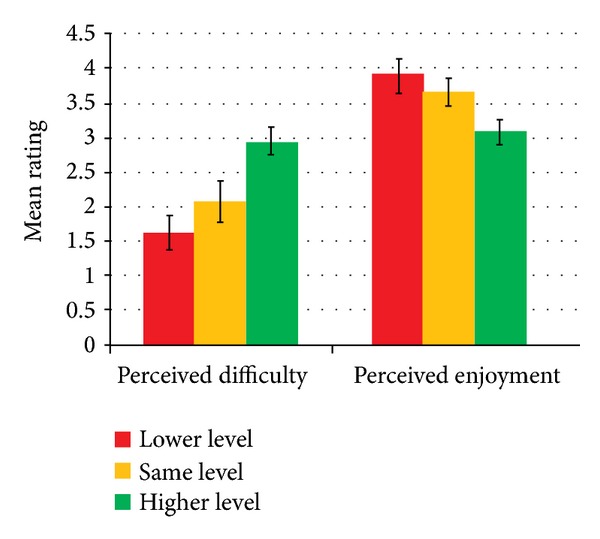
Patient's subjective rating with respect to adaptation.

Based on the patients' subjective responses, we calculated the average ratings of perceived difficulty and perceived enjoyment, for the three conditions of adaptation as shown by Figure 22. Kruskal-Wallis test showed that significant differences were found for* perceived difficulty* (H(2) = 13.062, *P* < 0.001; 1.64 for condition 1, 2.08 for condition 2, and 2.96 for condition 3) and* perceived enjoyment* (H(2) = 9.439, *P* < 0.05; 3.91 for condition 1, 3.67 for condition 2, and 3.09 for condition 3). This indicates that patients perceived the difficulty and enjoyment differently across the conditions of adaptation. Mann-Whitney pairwise comparison tests showed that patients perceived the training to be more difficult and less enjoyable when the training was adapted to a higher level compared to when they had to adapt to a lower level (*P* < 0.05).

The finding which showed that as the levels got more difficult the patients perceived the training to be less enjoyable is somewhat different than our previous finding where patients perceived the same high level of enjoyment despite the change in difficulty levels. We think that social effect plays a role in this case. In a collaborative training session, the patients might have felt more social pressure to perform as good as the training partner. They might have feared that they will hinder the collaboration if they perform less well. However, additional investigation is needed to confirm this thought. Another possible explanation is that the differences among the difficulty levels were quite prominent. This calls for a further investigation to find a subtler difference of difficulty that maintains the patient's enjoyment in training.

**Figure 23:**
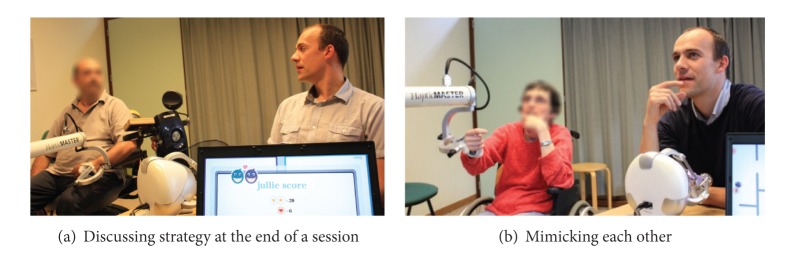
Different behaviors observed during the social maze game.

With regard to social interaction, we believe that the social maze game has become a social play medium between patients and their therapist. We have observed some relevant behaviors which indicated the development of social interaction during the collaborative rehabilitation training.  Figure 23 depicts two examples of behaviors shown by the pairs of participants throughout the training sessions. The most shown behavior during the social maze game was the act of discussing strategy (Figure 23(a)). It was pretty obvious that the nature of the training exercise requires the two participants to closely collaborate and discuss their strategy and necessary actions. This behavior happened throughout the whole session, sometimes followed by the act of looking at each other. At the beginning and during the session, participants discussed what actions they should perform and the best way to perform them. At the end of the session, participants briefly reviewed their previous session and how they should perform better on the next session. In some participants, we can also observe the behavior of unconsciously mimicking each other during the strategy discussion (Figure 23(b)). Throughout the sessions, we can observe that all participants showed other behaviors such as smiling, laughing, and chuckling. This mostly happened when one of them made an error such as encountering the devil or hitting the laser beam. A couple of participants tended to make jokes during the training session that resulted in both of them laughing at each other.

## 7. Discussion

Our work has presented an investigation to integrate adaptivity and personalization into individual rehabilitation training and collaborative rehabilitation training for MS patients. Typically rehabilitation training is individual, where a MS patient performs the training exercises alone under the supervision of a therapist. However, rehabilitation training can take place in a collaborative setting as well, where performing the training exercises involves more than only a single MS patient. By changing the nature of rehabilitation training to be collaborative, we can enhance the patient's motivation and also support social interaction between the patient and his/her training partner (e.g., a family member, friend, or therapist).

We have discussed and implemented the adaptation of automatic difficulty level adjustment in both individual training (i.e., the penguin painting game) and collaborative training (i.e., the social maze game). Two user studies have been carried out to investigate the outcome of this adaptation. Overall, we can conclude that providing adaptive difficulty in the training exercises has delivered an adaptive personalized training to each MS patient according to his/her own individual training progress. Patients and the therapist have appreciated the automatic adaptation of difficulty levels and considered it to provide more variety in the training and also give the patients more freedom to train on their own without any interference from the therapist to manually adjust the exercise parameters. This kind of adaptation could be useful to determine an appropriate level to start training on a certain day according to the patient's condition on that day, thus less determined by the previous training or the therapist.

Moreover, we have observed the development of social interaction between patients and their training partner during the course of the collaborative rehabilitation training exercises. While performing the social maze game collaboratively, they have showed several particular behaviors such as smiling, laughing or chuckling, looking at each other, and discussing strategy. We realized that the adaptation in the collaborative training exercise has been implemented with a focus on the patient's needs and characteristics despite the existence of a training partner. In the sympathetic social scenario, it may be interesting to investigate how or what kinds of adaptation can facilitate the family member or the therapist to achieve a more engaging and motivating collaboration with the patient. For the empathetic social scenario, we also need to investigate how the different types of adaptation can accommodate the needs and characteristics of another patient as a training partner.

It is not our focus to carry out an in-depth investigation on the adaptation algorithm used in these user studies. We were more interested to observe the patients' response with respect to the automatic adjustment of difficulty levels. We realize that a more accurate and well-defined algorithm could be provided. Therefore, further investigation is needed to optimize the adaptation algorithm which also matches the judgment of therapists on the trigger and timing of adaptation. Another next step is to extend our investigation of integrating adaptivity and personalization into MS rehabilitation training to other types of adaptation that may help patients during the course of their training, for example, automatically adjusting the assistance level based on the detected muscle fatigue. In some cases, the muscle fatigue might develop during long training; thus, it might be necessary to provide the patients with some assistance to help them perform the task and continue their training.

## 8. Conclusion

This paper has discussed our investigation on the notion of integrating adaptivity and personalization into individual and collaborative rehabilitation training for MS patients. We have strived for developing adaptive personalized training games by introducing the automatic adjustment of difficulty levels in the penguin painting game (individual training) and the social maze game (collaborative training). The user studies showed that none of the MS patients experienced the same training trajectory as the other. This confirms that every patient progresses differently, which encourages the need for adaptation. Adaptive personalized training, which was delivered to every MS patient according to his/her own individual training progress, has shown to be beneficial and much appreciated. The automatic adjustments of difficulty levels in the training games were also considered to provide more variety in the training and more freedom to train independently.

## Figures and Tables

**Table 1:** Overview of the penguin painting game parameters in the different difficulty levels.

Game parameter	Difficulty level						
	Level −3	Level −2	Level −1	Level 0	Level 1	Level 2	Level 3
Size of penguin	All small	90% small	70% small	50% small	30% small	10% small	All large
		10% large	30% large	50% large	70% large	90% large	
Speed of devil	Slow	Slow	Medium	Medium	Medium	Fast	Fast
Frequency of devil	Infrequent	Infrequent	Normal	Normal	Normal	Frequent	Frequent
Length of stabilization	Short	Short	Normal	Normal	Normal	Long	Long
Obstacle wall	No	No	No	No	No	Yes	Yes
Amount of coloring bucket	2	2	3	3	3	4	4
Width of coloring bucket	All wide	Bottom: 1 wide, 1 narrow	Bottom: 2 wide, 1 narrow	Bottom: 1 wide, 2 narrow	Bottom: 1 wide, 2 narrow	Bottom: 2 wide, 2 narrow	All narrow
		Top: 1 wide, 1 narrow	Top: 2 wide, 1 narrow	Top: 2 wide, 1 narrow	Top: 1 wide, 2 narrow	Top: All narrow	
Exit platform	No	No	No	No	Yes	Yes	Yes

**Table 2:** Personal information of MS patients in the user studies.

Patient	Gender	Age (years)	Diagnosis duration (years)	Training hand
1	Male	64	14	Left
2	Female	58	3	Left
3	Male	71	10	Right
4	Female	47	14	Right
5	Male	57	27	Left
6	Male	55	27	Left
7	Female	64	25	Right
8	Male	56	30	Left
9	Male	72	34	Left

**Table 3:** Clinical characteristics of MS patients in the user studies.

Patient	MI	ARAT	BFM-prox	BFM-dist
1	76	41	25	40
2	83	56	36	29
3	84	46	32	28
4	76	56	36	30
5	55	41	23	21
6	47	7	18	24
7	72	18	31	25
8	60	30	27	24
9	50	1	12	7

MI: motricity index (maximal score = 100); ARAT: action research arm test (maximal score = 57); BFM-prox: Brunnstrom Fugl-Meyer proximal score (maximal score = 66); BFM-dist: Brunnstrom Fugl-Meyer distal score (maximal score = 66).

**Table 4:** Overview of the social maze game parameters in the different difficulty levels.

Game parameter	Difficulty level							
	Level −3	Level −2	Level −1	Level 0	Level 1	Level 2	Level 3	Level 4
Viscosity of movement	Very low	Low	Low	Normal	Normal	High	High	Very high
Speed of devil	Slow	Slow	Slow	Medium	Medium	Fast	Fast	Fast
Length of laser beam	Short	Short	Short	Normal	Normal	Long	Long	Long
Friction of bomb	No	No	No	Less	Less	Much	Much	Much
Friction of wall	No	No	No	Less	Less	Much	Much	Much
